# The new WHO decision-making framework on vaccine use in acute humanitarian emergencies: MSF experience in Minkaman, South Sudan

**DOI:** 10.1186/s13031-018-0147-z

**Published:** 2018-03-26

**Authors:** Monica Rull, Sophie Masson, Nicolas Peyraud, Marco Simonelli, Alexandre Ventura, Claire Dorion, Francisco J. Luquero, Florent Uzzeni, Iza Cigleneki

**Affiliations:** 1 Medecins Sans Frontieres Switzerland, Geneva, Switzerland; 2 Epicentre, Paris, France; 3 Medecins Sans Frontieres, Juba, South Sudan

**Keywords:** South Sudan, Measles, Cholera, Respiratory infections, Diarrheal diseases, Vaccine preventable outbreaks, Safe drinking water, Crude mortality rate, Under 5 mortality rate

## Abstract

**Introduction:**

The main causes of death during population movements can be prevented by addressing the population’s basic needs. In 2013, the World Health Organization (WHO) issued a framework for decision making to help prioritize vaccinations in acute humanitarian emergencies. This article describes MSF’s experience of applying this framework in addition to addressing key population needs in a displacement setting in Minkaman, South Sudan.

**Case description:**

Military clashes broke out in South Sudan in December 2013. By May 2014, Minkaman, a village in the Lakes State, hosted some 85,000 displaced people. MSF arrived in Minkaman on 28 December 2013 and immediately provided interventions to address the key humanitarian needs (health care, access to drinking water, measles vaccination). The WHO framework was used to identify priority vaccines: those preventing outbreaks (measles, polio, oral cholera vaccine, and vaccine against meningococcal meningitis A (MenAfrivac®)) and those reducing childhood morbidity and mortality (pentavalent vaccine that combines diphtheria, tetanus, whooping cough, hepatitis B, and *Haemophilus influenzae* type B; pneumococcal vaccine; and rotavirus vaccine). By mid-March, access to primary and secondary health care was ensured, including community health activities and the provision of safe water. Mass vaccination campaigns against measles, polio, cholera, and meningitis had been organized. Vaccination campaigns against the main deadly childhood diseases, however, were not in place owing to lack of authorization by the Ministry of Health (MoH).

**Conclusions:**

The first field use of the new WHO framework for prioritizing vaccines in acute emergencies is described. Although MSF was unable to implement the full package of priority vaccines because authorization could not be obtained from the MoH, a series of mass vaccination campaigns against key epidemic-prone diseases was successfully implemented within a complex emergency context. Together with covering the population’s basic needs, this might have contributed to reducing mortality levels below the emergency threshold and to the absence of epidemics. For the WHO framework to be used to its full potential it must not only be adapted for field use but, most importantly, national decision makers should be briefed on the framework and its practical implementation.

## Background

By late 2016, more than 40 million people worldwide had been internally displaced, mainly as a result of conflicts. This is the highest number ever reported [1]. Overcrowding, high population density, rudimentary or inappropriate shelters, poor access to water and sanitation, and poor nutritional status due to food insecurity, combined with limited access to preventive and curative health care, increase the risk of the spread of infectious diseases. This can result in higher morbidity and mortality, especially in camp settings [2]. Improvements in the response to complex emergencies have focused mainly on the prevention and control of malnutrition and diseases with epidemic potential, leading to a decline in morbidity and mortality in camp settings when effective assistance is provided [3]. However, the main causes of death (typically diarrheal diseases, respiratory infections, measles, and malaria where endemic) are still common and can be easily prevented or treated [4].

Emergency humanitarian responses to situations involving displaced people are focused on preventing excess mortality by addressing essential needs based on the “MSF Top Ten Priorities” (Table 1) framework drawn up by Médecins Sans Frontières (MSF) in the late 1990s [5] or SPHERE standards [6].

**Table 1:** The Top Ten Priorities for Refugee Health

1.Initial assessment	Quantitative and qualitative information on background to the displacement, population, risk factors related to the main diseases and requirements in terms of resources through observation, interviews, sample surveys, mapping.
	Usually approximate, results may need to be corroborated later.
2.Measles immunization	Displacement, overcrowding and poor hygiene are factors that encourage emergence of large scale epidemics.
	Mass vaccination of children from 6 months to 15 years should be a priority during the first week.
3.Water and sanitation	Prevention of diarrhoeal diseases and survival
	Ensure immediate provision with temporary water supply until more permanent solutions (wells) can be found
	Indicators in regard to water supply and latrines must be monitored.
4.Food and nutrition	Malnutrition is often associated with displacement
	Provision of food ration to cover daily minimum needs
	Feeding programs for specific groups are supplementary feeding for moderately malnourished and therapeutic or intensive feeding for the severely malnourished.
5.Shelter and site planning	Provide protection from environment
	Prevent transmission of diseases with epidemic potential link to overcrowding and inadequate shelter
	Ensure sufficient infrastructure for providing services (e.g. health facilities)
6.Health care in emergency phase	Create a decentralized network of health facilities
	Provide manuals and guidelines for standardization
	Ensure medical material and drugs in sufficient quantity and quality – (i.e. Kits of essential drugs and material)
7.Control of communicable diseases and epidemics	Four greatest killers: measles, diarrhoea, acute respiratory infections and malaria
	Higher risk of communicable diseases: measles, cholera, shigellosis, meningitis etc.
	Preventative measures are to be privileged when possible (e.g. vaccination campaigns)
8.Public health surveillance	Monitoring the health status of the population
	Daily collection of selected health data – only cover diseases or other health problems that can be controlled by preventive or curative interventions.
	Most useful health indicator is the daily crude mortality rate
	Objectives: warn of an impending epidemic, monitor the main diseases occurring In the population and measure the impact of health programs
9.Human resources and training	Determine staff requirements after identification of activities
	Human resources management including recruitment and training
	Important to ensure the link with the community: Home visitors
10.Coordination	Must be organized at the onset of the crisis
	A good system involves: overall clear leadership with good communication lines and that overall policy is standardized

The intervention priorities in the emergency phase cover 10 sectors. Ideally these interventions should be carried out simultaneously

Adapted from: Refugee Health: An approach to emergency situations. Médecins Sans Frontières

In 2013, the World Health Organization (WHO) issued a framework for decision making to help prioritize vaccinations in acute humanitarian emergencies [7]. In addition to vaccination against measles (a major cause of death in humanitarian disasters [8] and part of a standard emergency response [5, 9]), the tool proposes a systematic framework to assess: 1) the risk of vaccine-preventable, outbreak-prone diseases such as polio, cholera, meningococcal meningitis, and hepatitis E; and 2) the main deadly childhood diseases, by including new and underutilized vaccines, notably pentavalent (diphtheria, tetanus, whooping cough, hepatitis B, *Haemophilus influenzae* type B), pneumococcal, and rotavirus vaccines. The decision-making process consists of three steps: 1) epidemiological risk assessment to determine and grade the risk of each vaccine-preventable disease (VPD); 2) assessment of the main characteristics of the vaccines (suitability, availability, affordability, etc.) and their amenability to mass vaccination campaigns; and 3) assessment of contextual considerations.

During the recent large-scale displacement in Minkaman, South Sudan, MSF used the WHO decision-making framework to define priority vaccinations in addition to addressing essential needs of the population. To our knowledge, this is the first utilization of the WHO framework. Here we describe our experience of applying it during an acute emergency and suggest some recommendations for future use.

## Case presentation

### Setting and context

After decades of civil war, South Sudan is still undergoing a political-military crisis with severe humanitarian consequences. South Sudan has some of the poorest health indicators in the world and is prone to outbreaks of communicable diseases. In addition, the health system has limited capacity to respond to emergencies without external aid; 80% of health care is provided by non-governmental organizations [10]. In 2013, almost half of the population lived below the poverty line, and only 57% of people had access to an improved water source [11].

**Fig. 1:**
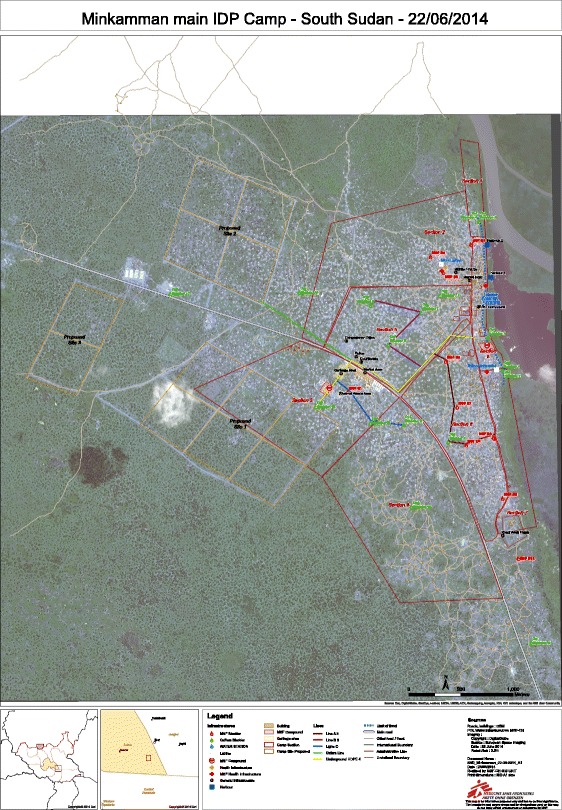
Minkaman map

In December 2013, armed conflict led to a massive population displacement in South Sudan. Many people fled fighting around the city of Bor in Jonglei State towards Minkaman – a village in South Sudan (Lakes State) located approximately 20 km south of Bor, on the river Nile. Minkaman itself was not directly affected by the violence (Fig. 1, map). Before the crisis, it had an estimated population of 7000 inhabitants. The influx of newly displaced people reached 85,000 in 5 months [12]. The displaced population was widely spread in a swamp-like area along the Nile and had limited access to clean water and sanitation, and the existing infrastructure was unable to meet the needs of the increasing population.

### Description of the intervention

The MSF team arrived in Minkaman on 28 December 2013. After meeting with the local authorities and other humanitarian actors present, the team immediately launched an emergency intervention focused on healthcare provision and access to safe drinking water. MSF did not prioritize food, distribution of non-food items, or delivery of shelter, as these were planned by other partners.

**Fig. 2:**
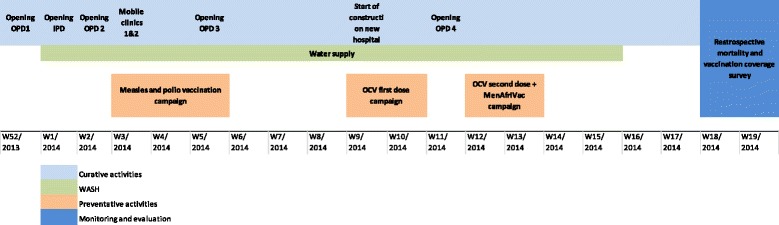
Chronology of MSF activities

A chronogram of MSF’s intervention is shown in Fig. 2. Primary health care was organized as soon as the team arrived, with two outpatient clinics being opened. Curative care was soon expanded, with hospitalization capacity and a nutritional program added. To address the high burden of watery diarrhea, several oral rehydration points were set up in the community and lasted until the end of March 2014, when the number of cases of watery diarrhea declined.

The water treatment station, water trucking, and bucket chlorination were functional within 3 days of MSF’s arrival. By 3 January 2014, five liters of treated water/per person/per day was being provided to the displaced population, an acceptable quantity in the initial phase of an emergency [8]. After a pipeline designed for adaptation to population movements was set up, the amount of water distributed doubled. The pipeline also reduced dependence on trucks (a costly solution unable to reach all sites in the rainy season). A community-based surveillance system for mortality was established. The internally displaced population camp was divided into eight sections (Fig. 1, map) covered by 60 community health workers (CHW) who collected weekly information about numbers of deaths from the heads of households and community leaders (without verbal autopsy). CHW also conducted active case finding for malnutrition (based on mid-upper arm circumference (MUAC) screening) and directed and referred sick patients to the health facilities.

By July 2014, more than 30 non-governmental organizations and several UN agencies were present in the area. Consequently, MSF decided to hand over its activities and close the Minkaman project on 15 October 2014.

### Prioritizing vaccines and use of WHO framework

**Fig. 3:**
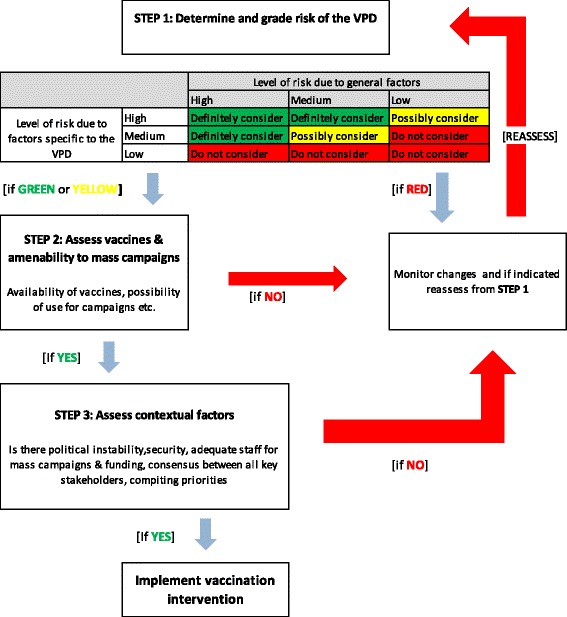
Decision making steps on vaccine use in humanitarian emergencies

We used the above mentioned WHO framework to define priority vaccinations to address the risk of vaccine-preventable outbreaks and VPD causing the highest morbidity and mortality. We used the three-step approach shown in Fig. 3. We assessed the general risks first and then the risk for each specific VPD. The major general risk factors were overcrowding and extremely precarious access to water and sanitation, in addition to high birth rates in a young population, moderately elevated malnutrition rates (a severe acute malnutrition rate of 0.5% and a moderate acute malnutrition rate of 2.7%, according to screening using MUAC tape (unpublished MSF program data)), and limited access to health care. In terms of specific diseases, we identified four high-risk, epidemic-prone VPD: measles, polio, cholera, and meningococcal meningitis. Measles was already addressed through a vaccination campaign organized within weeks of displacement, in line with existing defined priorities, and combined with polio vaccine (owing to recent outbreak of wild polio in Unity State of South Sudan). Cholera outbreaks were reported in several locations in South Sudan usually associated with the rainy season. Similarly, South Sudan has experienced large-scale epidemics of meningococcal meningitis during the dry winter months, and at the time of this intervention MenAfrivac® had not yet been introduced. In terms of the main childhood deadly diseases, all eight main VPD (measles, polio, diphtheria, tetanus, pertussis, pneumonia due to *Haemophilus influenzae* type b (HiB), streptococcal pneumonia, and rotavirus) were considered to be of high risk, owing to low population immunity (HiB, pneumococcal conjugate vaccine (PCV), and rotavirus vaccines had not yet been introduced in the country), overcrowding, a young population, and seasonality.

All vaccines assessed proved to be suitable for vaccination campaigns. Polio and MenAfriVac® were suitable for a one-round vaccination campaign. Measles, cholera, PCV, and rotavirus vaccine required two rounds, while pentavalent and hepatitis E vaccine required three rounds. In order to optimize the campaigns, we combined polio with measles vaccine in the first round, and MenAfriVaq® vaccine was administered together with the second dose of oral cholera vaccine (OCV).

Lastly, we looked at contextual factors that appeared favorable for vaccine use, including the feasibility of accessing the vaccines and implementing sequential campaigns in the closed setting of Minkaman. A proposal was made to the Ministry of Health (MoH) to provide oral cholera, MenAfriVac®, pentavalent, PCV, and rotavirus vaccines to the population in a series of vaccination campaigns combining different vaccines (Table 2).

**Table 2:** Risk analysis preventive vaccination, Minkaman

Considerations	Specific considerations						Assessment conclusion
Epidemiological/ risk assessment	General risk factors:		Disease-specific risk factors:				
	Limited access to curative health services. Young population and high birth rate. Overcrowding. Insufficient water, sanitation and hygiene		Low population immunity: High risk for meningitis and cholera (no previous vaccination, no large outbreak in the past 3 years), pneumococcal disease, HiB and rotavirus (not yet introduced in EPI)				Overall specific risk High/moderate High
							VPD high specific risk associated for:
							Measles, meningitis, cholera, polio, HiB, Pneumococcal disease and rotavirus
			High burden of disease: main child deadly diseases are respiratory tract infections and diarrhea. Seasonality Cold dry season				
Vaccine characteristics	Antigen	Type	Recommended dosage	VE 1 dose	Target pop	cm^3^/dose	
	Measles	Live attenuated	1 dose	~ 85%	> 6 m to 15y	0.75–5.22	Suitable for vaccination campaign two rounds (plus EPI)
	Cholera (oral Sanchol°)	Inactivated	2 doses	N/A	>_ 1y	16.8–24.4	Suitable for vaccination campaign two rounds
	Polio	Live attenuated	3 doses	~ 50%	6w to 5y	0.24–3.2	Suitable for vaccination campaign one round (plus EPI)
	PCV	Inactivated	2 doses	up to 70%	6w to 5y	4.8–15.7	Suitable for vaccination campaign two rounds (plus EPI)
	Pentavalent (DPT, HiB, Hep B)	Inactivated	3 doses	N/A	6w to 7y	2.6–5.1	Suitable for vaccination campaign three rounds (plus EPI)
	MenAfriVac® A	Inactivated	1 dose	~ 75–95%	1 to 29y	2.6	Suitable for vaccination campaign one round
	Hep E		3 doses	N/A	>16y	132.6	Suitable for vaccination campaign three rounds
	Rotavirus (Rotarix® liquid)		2 doses	N/A	6w to 2y	17,1	Suitable for vaccination campaign two rounds (plus EPI)
Contextual constraints and facilitators	Ethical	Political		Security	Economic/logistic constraints		
	No community opposition. Informed consent process possible at community and individual level. Target population displaced and host community for all vaccinations	Current EPI policy limiting immunization activities (no pentavalent, rotavirus, PCV). Measles, polio cholera and meningitis campaigns validated. Antecedent of cghPCV vaccination approved		The area of Minkaman is currently stable. No previous threats to immunization activities. No specific risk to health workers or those immunized	Funding available. Sufficient vaccine supply. Vaccination teams already identified and trained in both injectable and oral vaccines. Cold chain and infrastructure already available and in place		No major barriers for immunization activities.
							Further negotiation required to use antigens not yet included in the EPI.
Conclusion	In addition to mass vaccination campaigns targeting diseases with epidemic potential (measles, polio, meningitis and cholera), we propose a series of campaigns with new and underutilized vaccines (pentavalent, pneumococcal and rotavirus) targeting the most common childhood vaccine preventable diseases AND follow up with routine vaccination activities. We believe such vaccination campaign achieving high coverage in a displaced population can have a very important impact on childhood morbidity and mortality. The 3 rounds of campaigns necessary are feasible in this setting with logistic and human resources available.						

### Summary of key intervention results

From 1 January to 15 October 2014 (the period of MSF’s intervention), 52,047 people attended the outpatient clinics and 2032 patients were admitted to the inpatient department. Seven hundred thirty-seven children were included in the nutritional program (with 161 receiving intensive care), and 20,778 antenatal consultations took place. In addition, 48 million liters of safe water were distributed to the displaced population (unpublished MSF program data).

MSF conducted preventive vaccination campaigns against measles, polio, cholera, and meningitis A from January to March 2014. Measles vaccine was administered concomitantly with polio vaccine to 13,256 children aged under 5; 54,415 people were vaccinated against cholera, and 32,681 people aged 1–30 years were vaccinated against meningitis A. To monitor and evaluate the intervention, we conducted a cross-sectional population-based survey between 3 and 9 May 2014 to estimate vaccination coverage and examine mortality rates. Two stage cluster sampling was used.

Measles vaccination coverage was estimated at 73.9% (95%CI: 68.8–78.3%). OCV coverage was 65.5% (95%CI: 61.2–69.6%) for two doses and 84.1% (95%CI: 81.5–86.3%) for at least one dose (before the catch-up round). MenAfrivac® vaccination coverage was estimated at 77.3% (95%CI: 73.5–80.8%).

The overall retrospective crude mortality rate (CMR) during the study period (end December 2013 to end April 2014) was estimated at 0.59 deaths/10,000 people/day (95%CI: 0.43–0.82) and the under-5 mortality rate (U5MR) at 0.50 (95% CI: 0.43–0.82) (unpublished data from Epicentre). Thus, both CMR and U5MR were below the emergency threshold for sub-Saharan Africa (0.80/10,000/day and 2.1/10,000/day, respectively) [13].

## Discussion

The MSF intervention in Minkaman followed MSF’s “Ten Top Priorities”, which have been guiding its emergency interventions since the late 1990s. In addition, we used the new WHO framework for prioritizing vaccines in acute emergencies as a complementary tool, with the aim of rapidly reducing the risk of vaccine-preventable outbreaks and reducing the morbidity and mortality due to the main vaccine-preventable diseases during a period of extreme vulnerability.

The use of oral cholera vaccine has already been discussed in South Sudan, and several pre-emptive campaigns were planned in the same period [14]. Similarly, the preventive use of MenAfriVac® was quickly approved, and the vaccine could be delivered at the same time as the second dose of OCV.

We successfully organized a series of vaccination campaigns targeting outbreak-prone diseases (measles, polio, cholera, and meningococcal meningitis) in a complex emergency. This was the first time that the MenAfriVac® vaccine had been used in a pre-emptive mass campaign in a complex emergency, and the first time that it was delivered concomitantly with OCV. Although acceptable, the OCV coverage was lower than in some of the other campaigns taking place at the same time [14], probably at least partially owing to high mobility of the population; high population mobility can also partially explain the low coverage for other antigens. We demonstrated that, despite logistic constraints, conducting several mass vaccination campaigns in displacement situations is feasible, even within a highly mobile population as is the case in South Sudan. However, we were unable to obtain authorization from the MoH for use of vaccines against the main deadly childhood diseases (pentavalent, pneumococcal, and rotavirus vaccines). None of these vaccines had been introduced in a routine immunization program at the time in South Sudan, although preparations were ongoing for the introduction of pentavalent vaccine into the Expanded Program on Immunization. This might have contributed to the reluctance of health authorities, which prioritized routine activities over emergency mass campaigns. However, pentavalent and pneumococcal vaccines have both been previously used in complex emergency situations n South Sudan, although in a setting of Sudanese refugees in Yida [15]. It is also likely that MSF became less inclined to pursue negotiations once authorization had been obtained for organizing the prevention of outbreak diseases and the situation in Minkaman stabilized.

The use of the framework is complex and was not easy to interpret during our Minkaman intervention. The first part (epidemiological risk assessment) provides some quantitative guidance for decision making (Fig. 3), but step 2 and particularly step 3 are qualitative, which makes summarizing findings into decisions difficult. To be widely used as guidance in emergency situations by response teams, the tool needs to be reviewed and simplified. However, work is underway to improve the framework and provide implementation guidance [16], which will probably address this point. Currently, national authorities and partners in the field are not necessarily aware that the framework exists, which compromises its wider uptake, particularly during acute emergencies with limited time for introducing new strategies. WHO and other health actors must proactively introduce the framework to national health authorities at local, regional, and national level and conduct real-time practical exercises with the MoH and health partners if it is to be used to its full potential.

Closer collaboration between humanitarian actors such as MSF and WHO will be helpful when lobbying national authorities. Lastly, more flexibility from the MoH is needed when some of the vaccines have not yet been introduced in the EPI, to enable implementation of preventive campaigns in humanitarian emergencies.

## Conclusion

We describe the first field use of a new WHO framework for prioritizing vaccines in acute emergencies. Although we were unable to implement the full package of priority vaccines, we successfully implemented a series of mass campaigns against key epidemic-prone diseases in a complex emergency context. Together with covering the population’s basic needs, this might have contributed to maintaining mortality levels below the emergency threshold and to the prevention of epidemics. For the WHO framework to be used to its full potential, it has to be adapted for field use, but most importantly its existence must be disseminated to national decision makers.
